# Patients’ and Care Partners’ Experiences with Biomarker-Informed Evaluation for Cognitive Impairment

**DOI:** 10.1093/geroni/igaf122.2937

**Published:** 2025-12-31

**Authors:** Cristina de Rosa, Jeong Eun Kim, Joshua Grill, Jennifer Lingler

**Affiliations:** University of Pittsburgh, Pittsburgh, Pennsylvania, United States; University of Pittsburgh, Pittsburgh, Pennsylvania, United States; University of California, Irvine, Irvine, California, United States; University of Pittsburgh, Pittsburgh, Pennsylvania, United States

## Abstract

As biomarker-informed diagnoses for Alzheimer’s disease and related dementias move from occurring primarily in controlled research settings to widescale clinical practice, understanding experiences of individuals with cognitive impairment and their care partners will be essential to meeting their unique needs. The Patient And family member Reactions to biomarker-informed ADRD DiagnosEs (PARADE) study conducted focus group discussions (FGDs) with diverse patients and care partners, with primary analysis identifying broad categories of information and support needs. This secondary analysis emphasized an in-depth examination of the broader emotional and interpersonal context in which needs arise. In PARADE, virtual FGDs with trained facilitators following a semi-structured guide asked participants about information and support needs following amyloid PET testing. Secondary thematic analysis of verbatim transcripts included 9 focus group participants (mean age 65.2 years, 55.56% female, 4 patient-care partner dyads and 1 other care partner) and preliminarily identified three themes. Patients and care partners described emotional and personal contexts, like past experiences caring for a family member with dementia, that were integral to their perspectives. Participants shared information within dyads (e.g., attending support groups alone and bringing back information for spouse) and with others with similar experiences. Knowledge of needs and disease trajectory allowed participants to advocate for themselves and anticipate future needs, like considering long-term care. Findings may suggest potential antecedent and contextual factors can contribute to identifying and meeting needs for support and continuing care. Both individuals undergoing biomarker-informed evaluations and family care partners may benefit from inclusion in care and planning discussions.

